# Laparoscopic intracavitary catheterization combined with bleomycin quadruple sclerotherapy for retroperitoneal lymphatic malformations: a single-center case series

**DOI:** 10.3389/fsurg.2026.1811626

**Published:** 2026-05-29

**Authors:** Guanghua Zhang, Ming Sun, Hongxi Guo, Kai Zheng, Yu Chen, Hongqiang Bian, Haibin Wang, Jun Yang

**Affiliations:** 1 Department of General Surgery, Wuhan Children’s Hospital, Tongji Medical College, Huazhong University of Science and Technology, Wuhan, China; 2 Department of Ophthalmology, Wuhan Children’s Hospital, Tongji Medical College, Huazhong University of Science and Technology, Wuhan, China; 3 Department of Radiology, Wuhan Children’s Hospital, Tongji Medical College, Huazhong University of Science and Technology, Wuhan, China

**Keywords:** bleomycin, laparoscopy, minimally invasive surgery, retroperitoneal lymphatic malformation, sclerotherapy, surgical innovation

## Abstract

**Background:**

Retroperitoneal lymphatic malformations (rLMs) are rare congenital anomalies with deep, complex anatomy adjacent to vital structures. Traditional treatments (open surgery, percutaneous sclerotherapy) carry high complication and recurrence rates. This study evaluated laparoscopic intracavitary catheterization combined with bleomycin quadruple sclerotherapy for massive cystic/multiloculated rLMs, aiming to establish a standardized minimally invasive paradigm.

**Methods:**

We retrospectively analyzed 5 pediatric patients with massive rLMs treated at our institution (2022-2023) using this sequential strategy. Perioperative data (operation time, blood loss, cyst fluid volume), postoperative recovery (ambulation, oral intake, hospital stay), sclerotherapy outcomes, and long-term follow-up (recurrence, complications) were reviewed. Complications were formally graded using the Clavien-Dindo classification.

**Results:**

All 5 patients completed treatment without conversion to open surgery or adjacent organ injury. Mean operation time was 128 ± 25 min, blood loss 2 ± 2 mL, and cyst fluid aspiration 135 mL (80–200 mL). All ambulated within 6 h, resumed oral intake at 20 ± 3 h, and had a mean hospital stay of 12 ± 6 d. No patient required analgesic intervention. According to the Clavien-Dindo classification, one patient experienced a grade I complication (self-limiting abdominal discomfort), with no higher-grade complications observed. During a median follow-up of 46 months (43–48 months), all 5 children achieved clinical cure, defined as the absence of symptoms and complete radiographic resolution on imaging (ultrasound/CT), with no recurrence.

**Conclusion:**

Laparoscopic intracavitary catheterization combined with bleomycin quadruple sclerotherapy is a minimally invasive, effective, and safe strategy for massive rLMs. It overcomes limitations of traditional treatments with minimal trauma, precision, and a favorable complication profile. The standardized protocol is reproducible, offering a new minimally invasive option for managing this rare retroperitoneal anomaly.

## 1 Introduction

Retroperitoneal lymphatic malformations(rLMs) are rare congenital anomalies that result from aberrant embryonic development of the lymphatic system, accounting for less than 1% of all lymphatic malformations(LMs) (1, 2). Typically presenting as massive cystic or multiloculated fused masses within the retroperitoneum, these lesions lie in intimate proximity to critical structures such as the kidneys, ureters, abdominal aorta, and inferior vena cava (3, 4). Their insidious onset often means they remain asymptomatic until they achieve considerable size, leading to complications including abdominal distension, organ compression(e.g., hydronephrosis), infection, or hemorrhage (5, 6).Consequently, the management of rLMs, dictated by their complex anatomy and large volume, represents a significant surgical challenge (1, 3).

The conventional treatment landscape for rLMs is fraught with limitations. Open surgical resection, long regarded as the curative standard, is associated with significant trauma, a high risk of injury to adjacent organs, and extensive intraoperative dissection (7, 8). Incomplete resection frequently results in recurrence rates exceeding 50% (6, 9). Percutaneous sclerotherapy, as a minimally invasive alternative, is often hindered by blind puncture, uneven distribution of the sclerosant within multilocular lesions, and the risk of drug extravasation (10, 11). For retroperitoneal lesions specifically, the percutaneous approach carries an elevated risk of intestinal perforation, particularly when the lesions abut the intestinal wall (10, 12).

In recent years, laparoscopic minimally invasive technology and image-guided sclerotherapy have emerged as promising treatments for abdominal and retroperitoneal cystic anomalies (13, 14). Bleomycin, a classic sclerosing agent, has demonstrated efficacy in treating large cystic LMs, with reported volume reduction rates exceeding 70% (15, 16). However, bleomycin monotherapy can induce substantial local inflammatory reactions and carries a dose-dependent, albeit low, risk of pulmonary toxicity (16, 17). To mitigate these issues, previous studies have suggested that formulating bleomycin with anti-inflammatory and analgesic adjuvants can enhance its local safety profile and patient tolerability (18, 19). Despite these advances, a standardized minimally invasive protocol that effectively integrates the precision of surgical visualization with optimized, controlled sclerotherapy to address massive, complex rLMs remains an unmet clinical need.

To address this gap, our team developed and implemented a sequential treatment strategy combining laparoscopic intracavitary catheterization with perioperative bleomycin quadruple sclerotherapy. This technique is designed to leverage the magnified, precise visualization afforded by laparoscopy to ensure accurate catheter placement and septal lysis, followed by controlled, sustained administration of a rationally formulated sclerosant. This study presents a retrospective analysis of five pediatric patients with massive rLMs treated with this innovative approach, with the primary aim of evaluating its feasibility, safety, and efficacy, and to establish a potential standardized paradigm for managing this challenging condition.

## 2 Materials and methods

### 2.1 Study design and participants

This single-center retrospective study was conducted at Wuhan Children's Hospital. The study protocol was approved by the Institutional Ethics Committee of Wuhan Children's Hospital(Approval No: 2026R022-E01) and was conducted in accordance with the ethical principles of the Declaration of Helsinki. Written informed consent, including permission for the publication of anonymized clinical and imaging data, was obtained from the legal guardians of all participants.

We included pediatric patients diagnosed with massive cystic or multiloculated retroperitoneal lymphatic malformations(rLMs) between January 2022 and December 2023, who underwent the described technique of laparoscopic intracavitary catheterization combined with bleomycin quadruple sclerotherapy. The diagnosis of rLM was confirmed by abdominal ultrasound and contrast-enhanced computed tomography(CT) or magnetic resonance imaging(MRI), consistent with the International Society for the Study of Vascular Anomalies(ISSVA) classification (1, 20).

**Inclusion criteria** were:(1) diagnosis of massive cystic or multiloculated rLM (maximum diameter≥10 cm); (2) no contraindications to laparoscopic surgery or bleomycin sclerotherapy;(3) availability of complete clinical, operative, and follow-up data.

**Exclusion criteria** were:(1) microcystic or diffuse infiltrative rLMs;(2) concurrent severe systemic infection or major organ dysfunction;(3) known allergy to bleomycin or any component of the quadruple sclerosant;(4) incomplete clinical or follow-up records.

### 2.2 Outcome measures and data collection

The primary outcome was clinical cure, defined as the absence of disease-related symptoms and complete radiographic resolution of the lesion on follow-up imaging (ultrasound or CT). Secondary outcomes included perioperative metrics, postoperative recovery parameters, and treatment-related complications.

Data were retrospectively collected from electronic medical records and included: (a)patient demographics; (b) clinical presentation; (c) preoperative imaging characteristics; (d) intraoperative data(operation time, estimated blood loss, volume of aspirated cyst fluid); (e) postoperative recovery(time to ambulation, time to resume oral intake, length of hospital stay); (f) details of sclerotherapy(number of sessions); (g) imaging outcomes{lesion volume reduction rate at 1 and 3 months postoperatively, calculated as:[(Preoperative maximum diameter- Postoperative maximum diameter)/ Preoperative maximum diameter] x100%}; and (h) long-term follow-up outcomes(recurrence, complications). All complications were prospectively documented and formally graded according to the Clavien-Dindo classification system[3].

Histopathological confirmation of lymphatic malformation was obtained in all cases via biopsy during surgery, defined by cyst walls lined by a single layer of endothelial cells and cavities filled with lymphatic fluid (4).

### 2.3 Surgical technique and sclerotherapy protocol

#### 2.3.1 Stage 1: Laparoscopic intracavitary catheterization

**Figure 1:**
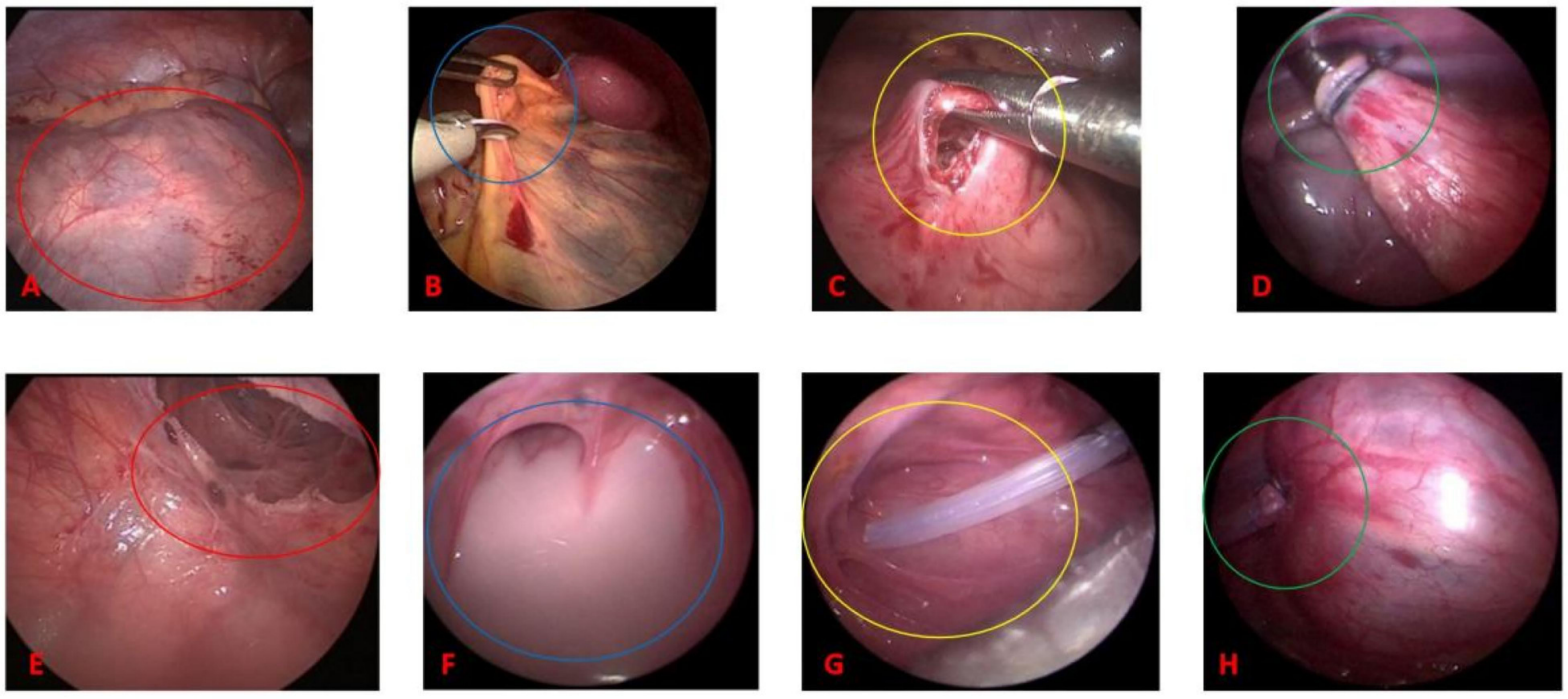
Schematic diagram of the surgical procedure:**(A)**∼**(H)**. **(A)** Laparoscopic view of the retroperitoneal cystic mass (red circle). **(B)** Meticulous dissection of the retroperitoneal tissue using an ultrasonic scalpel to expose the cyst wall (blue circle). **(C)** An incision is made on the cyst wall, and tissue is obtained from the edge of the incision for pathological biopsy (yellow circle). **(D)** The laparoscopic camera is introduced into the cyst cavity through a trocar, and the trocar is secured with sutures (green circle). **(E)** Under direct visualization, intracystic septa are divided through another incision using an ultrasonic scalpel (Red circle). **(F)** Aspiration of the cystic fluid, with the total volume recorded (blue circle). **(G)** A 12F silicone drainage tube is placed into the cyst cavity (yellow circle). **(H)** The drainage tube is secured with sutures at both the cyst wall incision and the skin exit site (green circle).

A standardized laparoscopic procedure was performed on all patients, with the surgical steps tailored to the anatomical specificities of retroperitoneal rLMs. The technique is summarized in Figure 1.

① Anesthesia and Perioperative Monitoring: Venous combined general anesthesia was used. Urinary catheters were indwelled for operations expected to last more than 2 h. Vital signs and end-tidal carbon dioxide partial pressure were continuously monitored intraoperatively to ensure safety; the surgical and anesthesia teams communicated in real-time to dynamically adjust anesthesia depth and optimize perioperative management.
② Surgical Position and Trocar Placement: Patients were placed in the supine position, with the monitor at the head end. A 5 mm incision was made at the umbilicus, a Trocar was inserted to establish pneumoperitoneum, and the pressure was maintained at 8–10 mmHg. Under laparoscopic surveillance, two additional 5 mm Trocars were inserted at the left and right lower abdomen corresponding to the surface projection of the lesion (total of 3 Trocars). A 30° laparoscopic lens was inserted through the umbilical Trocar, and 5 mm ultrasonic scalpel and forceps were inserted through the other two Trocars for dissection and traction. The exclusive use of 5-mm Trocars minimized abdominal wall trauma, aligning with the principles of minimally invasive surgery.
③ Laparoscopic Exploration and Lesion Dissection: Laparoscopic exploration was performed to clarify the location, shape of the lesion, and anatomical relationship with critical structures such as the kidneys, ureters, and abdominal aorta, and to assess surgical difficulty. The retroperitoneal space was dissected along the edge of the lesion using an ultrasonic scalpel to gradually expose the cyst wall, with strict protection of adjacent blood vessels and organs to avoid iatrogenic injury. Minor bleeding from small blood vessels was directly coagulated with an ultrasonic scalpel without additional ligation, reducing intraoperative trauma.
④ Intracystic Septum Lysis and Tissue Biopsy: Two 5 mm incisions were made at appropriate positions on the cyst wall, and a 5 mm × 5 mm lesion tissue was excised from the incision edge for pathological examination to confirm the diagnosis. A laparoscopic lens was inserted into the cyst cavity through one incision and fixed with 4-0 absorbable sutures to prevent displacement; an aspirator and ultrasonic scalpel were inserted through the other incision to completely lyse all intracystic septa under direct laparoscopic vision (care was taken to protect the outer cyst wall to avoid cyst fluid extravasation), and all cyst fluid was aspirated and the volume recorded (80–200 mL, mean 135 mL). This step overcomes a key limitation of percutaneous sclerotherapy: the ineffective treatment of intracystic septa. Complete lysis of intracystic septa ensures sufficient contact between the sclerosing agent and the cyst wall, improving treatment efficacy.
⑤ Intracavitary Drainage Tube Placement and Fixation: A 12F silicone drainage tube was inserted into the cyst cavity through the incision, and the indwelling depth was adjusted to 3–5 cm according to the lesion size to ensure sufficient contact with the cyst wall and avoid injury to adjacent organs. The cyst wall incision and drainage tube were sutured and fixed with 1-2 stitches of 2-0 absorbable sutures to prevent tube dislodgement; the other end of the drainage tube was drawn out through the left lower abdominal Trocar hole and fixed to the skin with 2-0 silk sutures to ensure unobstructed drainage. Finally, 20–50 mL of normal saline was injected through the drainage tube to check for leakage, and aspiration was performed to confirm unobstructed drainage.
⑥ Intraoperative Sclerotherapy and Closure: The pre-mixed bleomycin quadruple sclerosant (see 2.4 for composition) was slowly injected through the catheter, which was then clamped for 30 min. The operative field was inspected, pneumoperitoneum released, and port sites closed.

#### 2.3.2 Stage 2: Postoperative sclerotherapy via indwelling catheter

①Sclerosant Formulation: The bleomycin quadruple sclerosant was prepared fresh for each session. Its composition and rationale were: bleomycin (1 mg/mL, core sclerosing agent), iohexol (for radiographic monitoring of distribution), dexamethasone(to mitigate local inflammatory response), and lidocaine(for analgesia) (18, 19).
②**Treatment Sessions:** The first postoperative sclerotherapy session was performed 4–7 days after surgery. Residual fluid was aspirated, and 2–5 mL of the sclerosant was injected via the catheter, which was then clamped for 2 h.
③**Treatment Course:** Sclerotherapy was repeated at 1-2 week intervals if imaging indicated a residual lesion volume > 10%. The catheter was removed when clinical symptoms resolved, imaging confirmed lesion disappearance or residual volume < 10%, and the 24-hour drainage output was < 5 mL.

### 2.4 Follow-up

Patients were scheduled for follow-up at 1,3,6, and 12 months postoperatively, and annually thereafter. Each visit included clinical assessment and abdominal ultrasound. Contrast-enhanced CT was performed at 1,3, and 12 months to objectively evaluate lesion resolution. A chest CT was performed at 3 months to screen for potential bleomycin-induced pulmonary fibrosis (16, 17).

### 2.5 Statistical analysis

Given the rarity of the condition and the consequent small sample size (*n* = 5), this study is primarily descriptive. All data are presented using descriptive statistics only. Continuous variables are expressed as mean ± standard deviation or median(range), as appropriate. Categorical variables are presented as counts(n) and percentages. No inferential statistical tests were applied, as they are not appropriate for a case series of this size. Data management was performed using SPSS software (version 25.0; IBM Corp., Armonk, NY, USA).

## 3 Results

### 3.1 Baseline demographic and clinical characteristics of patients

A total of 5 pediatric patients (4 males, 1 female) with massive retroperitoneal lymphatic malformations(rLMs) were included. The median age was 7 years (range: 2–11 years), and the median weight was 24.0 kg (range: 13–33 kg). The mean maximum lesion diameter on preoperative imaging was 14 ± 3 cm (range: 10–16 cm). Clinical presentations included recurrent abdominal pain (*n* = 2), a palpable abdominal or inguinal mass (*n* = 2), and bilateral ureteral compression with mild hydronephrosis (*n* = 1). The baseline clinical and imaging characteristics are summarized in Table 1. Histopathological examination of intraoperative biopsies confirmed the diagnosis of lymphatic malformation in all cases.

**Table 1:** Clinical and imaging characteristics of the five pediatric patients with massive retroperitoneal lymphatic malformations.

Case no.	Sex	Age (years)	Weight (kg)	Maximum lesion diameter (cm)	Clinical presentation
1	Male	7	24	14	Recurrent abdominal pain
2	Male	9	29	16	Abdominal and inguinal mass
3	Male	2	13	10	Bilateral ureteral compression, mild hydronephrosis
4	Female	5	18	13	Recurrent abdominal pain
5	Male	11	33	14	Abdominal mass

All lesions were confirmed as cystic/multilocular lymphatic malformations on histopathology.

### 3.2 Perioperative and immediate postoperative outcomes

All five surgical procedures were completed laparoscopically without conversion to open surgery. There were no intraoperative complications, such as injury to adjacent organs, significant hemorrhage, or sclerosant extravasation. The mean operation time was 128 ± 25 min. The mean intraoperative blood loss was 2 ± 2 mL, and the mean volume of cyst fluid aspirated was 135 mL (range: 80–200 mL). All patients ambulated within 6 h postoperatively. The mean time to resume oral intake was 20 ± 3 h. The mean length of hospital stay was 12 ± 6 days. The detailed perioperative data are presented in Table 2.

**Table 2:** Perioperative and sclerotherapy outcomes.

Case no.	Operation time (min)	Blood loss (mL)	Cyst fluid aspirated (mL)	Time to ambulation (h)	Time to oral intake (h)	Hospital stay (d)	Postop sclerotherapy sessions (n)	Lesion reduction rate (1 mo)	Lesion reduction rate (3 mo)
1	135	1	150	<6	20	10	2	100%	100%
2	165	5	200	<6	26	21	3	77%	100%
3	100	1	80	<6	18	7	3	85%	100%
4	120	2	120	<6	19	9	2	100%	100%
5	118	2	125	<6	19	15	2	100%	100%
Mean ± SD	128 ± 25	2 ± 2	135 ± 46	<6	20 ± 3	12 ± 6	2 ± 1	92% ± 11%	100%

### 3.3 Complications according to the Clavien-Dindo classification

All postoperative adverse events were prospectively recorded and formally graded using the Clavien-Dindo classification system. One patient experienced grade I complications, presenting as mild self-limiting abdominal pain and vomiting on postoperative day 6, which resolved completely with dietary adjustment and did not require pharmacological intervention. No complications of grade II or higher (including high-grade fever > 38.5 °C, incision infection, lymphatic leakage, intestinal perforation, or symptoms suggestive of pulmonary fibrosis) were observed in any patient during the hospital stay. A chest CT scan performed at 3 months postoperatively ruled out bleomycin-induced pulmonary fibrosis in all cases.

### 3.4 Sclerotherapy efficacy and short-term radiographic outcomes

All patients completed the full course of postoperative sclerotherapy via the indwelling catheter. Three children required 2 sclerotherapy sessions, while the other two received 3 sessions. Follow-up imaging at 1 month postoperatively showed a mean lesion volume reduction rate of 92% ± 5%. By the 3-month follow-up, imaging (ultrasound or CT) confirmed complete radiographic resolution, defined as the absence of any visible cystic lesion in the original rLM location, in all 5 patients, corresponding to a 100% reduction rate.

### 3.5 Long-term follow-up outcomes and clinical cure

During a median follow-up duration of 46 months (range: 43–48 months), all 5 children remained asymptomatic. They all met the pre-defined criteria for clinical cure, which is the absence of disease-related symptoms combined with sustained complete radiographic resolution on follow-up imaging, with no evidence of recurrence. The single patient with preoperative bilateral hydronephrosis showed complete resolution of ureteral compression and normalization of the renal collecting systems on serial imaging.

### 3.6 Representative case

**Figure 2:**
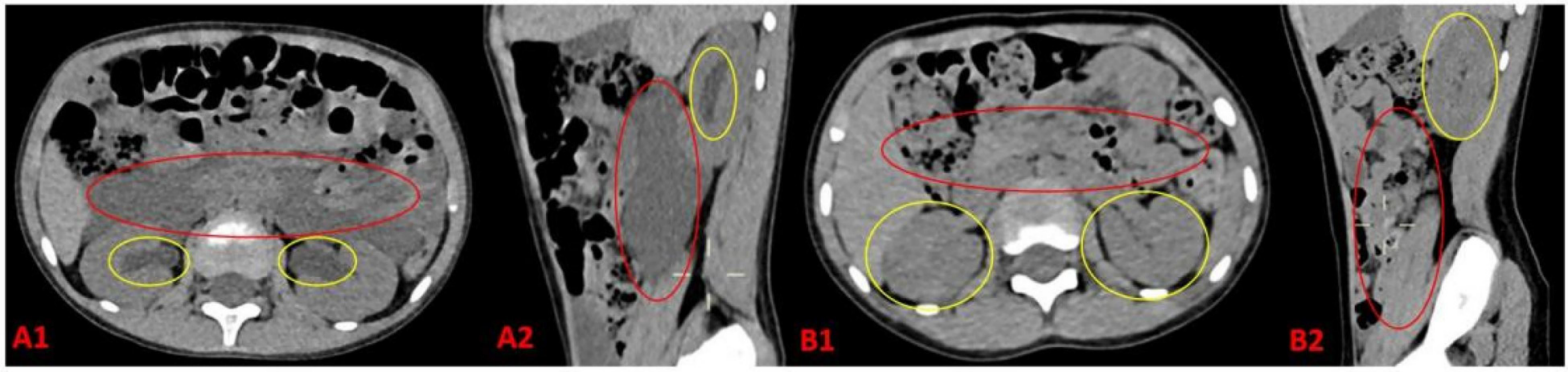
Postoperative follow-up imaging of a representative case. **(A)** Preoperative CT scans (axial, A1; sagittal, A2) show a large cystic lesion in the retroperitoneum (red circle) accompanied by bilateral hydronephrosis (yellow circle). **(B)** Postoperative CT scans at 3 months (axial, B1; sagittal, B2) demonstrate complete resolution of both the cystic lesion (red circle) and the bilateral hydronephrosis (yellow circle).

A representative case involved a 4-year-old boy with a massive rLM (maximum diameter: 16 cm) causing bilateral hydronephrosis (Figure 2A). He underwent successful laparoscopic intracavitary catheterization with intraoperative sclerotherapy, followed by 3 postoperative sclerotherapy sessions. Follow-up CT at 1 month showed marked reduction in lesion size and resolution of right-sided hydronephrosis. CT at 3 months (Figure 2B) confirmed complete disappearance of the rLM lesion and normalization of both renal collecting systems. At the latest follow-up (47 months), the patient was asymptomatic with no clinical or imaging evidence of recurrence.

## 4 Discussion

Retroperitoneal lymphatic malformations(rLMs) present a formidable surgical challenge due to their deep-seated location, complex anatomy adjacent to vital structures, and frequent presentation as large, multiloculated lesions. The conventional therapeutic paradigm, oscillating between maximally invasive open resection and minimally invasive yet sometimes imprecise percutaneous sclerotherapy, often forces a trade-off between completeness of treatment and procedural morbidity (7, 10, 11). This study introduces a novel, integrated strategy that sequentially combines laparoscopic intracavitary catheterization with bleomycin quadruple sclerotherapy. Our results, demonstrating a 100% clinical cure rate with no major complications or recurrences at a median follow-up of 46 months, suggest that this approach may effectively reconcile the efficacy-safety dichotomy inherent in managing massive rLMs. The following discussion contextualizes these findings, elucidates the technical and pharmacological rationale behind the observed outcomes, compares them with established modalities, and candidly acknowledges the study's limitations.

### 4.1 Interpretation of key outcomes: efficacy, safety, and long-term durability

The primary endpoints of any rLM treatment are complete and durable ablation of the lesion with minimal morbidity. In this small yet meticulously followed cohort, all five patients achieved clinical cure, defined as the absence of symptoms coupled with complete radiographic resolution. The absence of recurrence during a median follow-up exceeding 3.5 years is particularly significant, as congenital lymphatic anomalies are known for their potential for late recurrence (5, 21). This durable efficacy was achieved with an exemplary safety profile. The meticulous application of the Clavien-Dindo classification confirmed the absence of any major (Grade ≥ II) complications (3). The single recorded Grade I complication (self-limiting abdominal discomfort) resolved without intervention, underscoring the procedure's tolerability. Notably, feared complications such as intestinal perforation (a risk of percutaneous approaches) or symptomatic pulmonary fibrosis (a dose-dependent risk of bleomycin) were not observed (10, 15, 17). These outcomes collectively establish a strong foundation for considering this technique as a viable primary intervention for selected cases.

### 4.2 Comparative analysis with established treatment modalities

The favorable outcomes observed in our series gain further significance when contrasted with the historical limitations of conventional therapies. A systematic comparison is warranted to delineate the potential advantages of our integrated approach.

#### 4.2.1 Versus open surgical resection

Open resection, while offering the possibility of *en bloc* removal, is inherently traumatic. The extensive retroperitoneal dissection required often leads to significant blood loss, prolonged postoperative ileus, extended hospital stays, and risks injury to adjacent organs (e.g., ureters, vessels) and lymphatic leakage (6–8). In stark contrast, the mean intraoperative blood loss in our series was a negligible 2 mL, epitomizing the minimally invasive ethos (8, 13, 14). Laparoscopic visualization provides magnified, panoramic views of the retroperitoneum, allowing for precise identification and preservation of critical anatomy, thereby mitigating the risk of iatrogenic injury (7, 13, 14). Most importantly, by employing intracavitary sclerotherapy as the definitive ablative modality, our technique obviates the need for hazardous dissection and *en bloc* resection of the cyst wall from adherent structures. This fundamental shift from “resection” to “access and ablation” may explain the high efficacy without the morbidity profile of open surgery.

#### 4.2.2 Versus percutaneous sclerotherapy

Percutaneous sclerotherapy, though less invasive than open surgery, suffers from the critical limitation of being a “blind” procedure. For multiloculated rLMs, percutaneous needle placement may not access all septated compartments, leading to uneven sclerosant distribution, incomplete treatment, and high recurrence rates necessitating multiple sessions (10, 11). Furthermore, the “blind” puncture carries a non-trivial risk of bowel injury or sclerosant extravasation, which can result in peritonitis or intestinal perforation-a particularly grave concern for retroperitoneal lesions abutting the bowel (10, 12).

Our technique directly addresses these shortcomings. Laparoscopic intracavitary visualization enables direct inspection and complete lysis of all internal septa, transforming a complex multiloculated cyst into a single communicating cavity. This ensures uniform contact between the sclerosant and the entire endothelial lining, a prerequisite for effective ablation (15, 16). The securely placed indwelling catheter then serves as a dedicated, safe conduit for repeated drug administration, eliminating the risks associated with multiple percutaneous punctures. This controlled, targeted delivery system likely contributed to the high success rate with a mean of only 2.4 sclerotherapy sessions, potentially reducing the cumulative procedural risk and patient burden compared to standard percutaneous protocols (10, 18, 22).

#### 4.2.3 In the context of emerging systemic therapy (e.g., sirolimus)

The advent of sirolimus, an mTOR inhibitor, has provided a valuable systemic option for complex, diffuse, or surgically recalcitrant lymphatic anomalies (23, 24). However, its role typically involves long-term administration with associated monitoring for potential adverse effects (e.g., myelosuppression, hyperlipidemia) (23, 24). For well-demarcated, massive macro cystic rLMs as described in this series, our technique offers a potentially curative local intervention with a finite treatment duration. The two approaches are not mutually exclusive but could be complementary. A multimodal strategy, wherein laparoscopic catheterization and sclerotherapy are used to debulk a dominant macro cystic component, while adjuvant sirolimus manages residual microcystic disease, represents a promising frontier for complex cases (23, 24).

### 4.3 Rationale for innovation: technical and pharmacological synergy

The novelty and apparent success of this strategy stem from the synergistic integration of a refined surgical access technique with a rationally designed pharmacological regimen.

#### 4.3.1 The "access-for-ablation” paradigm

The core technical innovation is the repurposing of laparoscopy. Instead of attempting complex and risky resection, laparoscopy is used to achieve controlled, precise access to the cyst cavity. This allows for under-vision septal lysis, biopsy, and-most crucially-the secure placement of an indwelling catheter. This establishes a reliable “working port” for subsequent ablative therapy, a concept we term the “access-for-ablation” paradigm. This paradigm shift is key to overcoming the blindness of percutaneous methods while avoiding the trauma of open surgery (7, 13, 14, 16).

#### 4.3.2 Rational design of the quadruple sclerosant

The pharmacological innovation lies in the quadruple sclerosant formula, specifically designed to enhance efficacy and safety beyond bleomycin monotherapy. Each component serves a distinct, synergistic purpose: 1) Bleomycin acts as the core sclerosing agent, inducing endothelial fibrosis (15, 17). 2) Iohexol, a contrast agent, allows for radiographic confirmation of sclerosant distribution within the cavity, ensuring no leakage and complete filling (18). 3) Dexamethasone is incorporated to mitigate the intense local inflammatory response often triggered by bleomycin, thereby reducing postoperative pain, fever, and tissue edema (18, 19). 4) Lidocaine provides immediate local analgesia, improving patient comfort during and after the sclerotherapy session (19). This rational combination aims to maximize ablative effect while proactively managing its side effects, addressing a key limitation of single-agent sclerotherapy.

### 4.4 Study limitations and future directions

We acknowledge several limitations inherent in this preliminary study, which are crucial for contextualizing our findings. First, the single-center, retrospective design and small sample size (*n* = 5), while a reflection of the extreme rarity of massive rLMs (1, 2), constrain the statistical power and generalizability of our conclusions and introduce the potential for selection bias. Second, the absence of a concurrent control group treated with alternative modalities (e.g., open resection or standard percutaneous sclerotherapy) precludes definitive comparative efficacy claims; our favorable comparisons are necessarily drawn from historical literature. Third, although the median follow-up of 46 months is encouraging, longer-term surveillance is mandatory to confirm the durability of the cure, as very late recurrences have been reported in vascular anomalies (5, 21). Finally, this technique has specific anatomical indications; it is optimally suited for macro cystic or multiloculated lesions amenable to catheter placement and is not suitable for diffuse, microcystic, or infiltrative rLMs, which may be better addressed with pharmacotherapy (23, 24).

Future research should aim to overcome these limitations. Prospective, multi-center studies with larger patient cohorts are needed to validate our results and enable more robust comparisons with standard treatments (1–3). Technical refinements, such as optimizing sclerosant formulations or exploring the use of biodegradable materials, could further enhance the protocol (18, 19). As noted, investigating combination strategies with systemic agents like sirolimus for mixed-type lesions is a promising direction (23, 24). Ultimately, a deeper understanding of the molecular pathophysiology of rLMs may yield biomarkers predictive of treatment response, paving the way for personalized management strategies (19, 25).

## 5 Conclusion

In conclusion, the sequential strategy of laparoscopic intracavitary catheterization combined with bleomycin quadruple sclerotherapy presents a novel and promising minimally invasive paradigm for the management of massive, septated retroperitoneal lymphatic malformations. By synergistically combining the precision of laparoscopic access with the efficacy of a rationally formulated, targeted sclerotherapy, this approach appears to effectively balance the dual imperatives of radical treatment and minimal morbidity. Our initial experience, demonstrating a high rate of durable clinical cure with an excellent safety profile, suggests that this standardized protocol is both feasible and reproducible. It may thus fill a significant therapeutic gap for this challenging condition. Further validation through larger-scale, comparative prospective studies is now warranted to definitively establish its role in the clinical management algorithm for rLMs.

## Data Availability

The original contributions presented in the study are included in the article/Supplementary Material, further inquiries can be directed to the corresponding authors.
